# Influence of inverse dynamics methods on the calculation of inter-segmental moments in vertical jumping and weightlifting

**DOI:** 10.1186/1475-925X-9-74

**Published:** 2010-11-17

**Authors:** Daniel J Cleather, Anthony MJ Bull

**Affiliations:** 1 St. Mary's University College and Department of Bioengineering, Imperial College London, UK; 2 Department of Bioengineering, Imperial College London, UK

## Abstract

**Background:**

A vast number of biomechanical studies have employed inverse dynamics methods to calculate inter-segmental moments during movement. Although all inverse dynamics methods are rooted in classical mechanics and thus theoretically the same, there exist a number of distinct computational methods. Recent research has demonstrated a key influence of the dynamics computation of the inverse dynamics method on the calculated moments, despite the theoretical equivalence of the methods. The purpose of this study was therefore to explore the influence of the choice of inverse dynamics on the calculation of inter-segmental moments.

**Methods:**

An inverse dynamics analysis was performed to analyse vertical jumping and weightlifting movements using two distinct methods. The first method was the traditional inverse dynamics approach, in this study characterized as the 3 step method, where inter-segmental moments were calculated in the local coordinate system of each segment, thus requiring multiple coordinate system transformations. The second method (the 1 step method) was the recently proposed approach based on wrench notation that allows all calculations to be performed in the global coordinate system. In order to best compare the effect of the inverse dynamics computation a number of the key assumptions and methods were harmonized, in particular unit quaternions were used to parameterize rotation in both methods in order to standardize the kinematics.

**Results:**

Mean peak inter-segmental moments calculated by the two methods were found to agree to 2 decimal places in all cases and were not significantly different (p > 0.05). Equally the normalized dispersions of the two methods were small.

**Conclusions:**

In contrast to previously documented research the difference between the two methods was found to be negligible. This study demonstrates that the 1 and 3 step method are computationally equivalent and can thus be used interchangeably in musculoskeletal modelling technology. It is important that future work clarifies the influence of the other inverse dynamics methods on the calculation of inter-segmental moments. Equally future work is needed to explore the sensitivity of kinematics computations to the choice of rotation parameterization.

## Background

Inverse dynamics methods are commonly used for calculating inter-segmental forces and moments in linked chains of rigid segments. In biomechanics, chains of rigid linked segments are often used to model the human body and in particular the upper or lower extremities. To this end, inverse dynamics methodologies have been widely employed to analyze human movement [1]. The inverse dynamics method is based upon measuring the forces and moments on the most distal end of a linked chain of rigid body segments in combination with an assessment of the 3D kinematics of these segments. The Newton-Euler equations of motion can then be applied to each segment in turn, moving from distal to proximal up the kinetic chain in order to evaluate the inter-segmental forces and moments.

Dumas et al. [2] have recently identified four distinct inverse dynamics methods within the bioengineering literature. According to Dumas et al., the most frequently employed methodology employs vectors and Euler angles. This method is based upon using the Euler angles to derive the kinematics of the movement, calculating the force vectors at each joint in the laboratory fixed global coordinate system (GCS) and the moment vectors in the body fixed local coordinate system [LCS; 1]. An alternative method identified by Dumas et al. includes a formalism that is based upon using unit quaternions and wrench notation and that was first proposed by the authors themselves [3]. Quaternions are a mathematical construct that can be used both to parameterize and compute rotations, and have been shown to represent the most economical non-singular parameterization of rotation [4], and are consequently employed within the method to compute the kinematics. Wrenches are used to describe both forces and moments in the GCS which facilitates the computation of the inverse dynamics within the GCS alone.

The wrench notation and unit quaternion method of Dumas et al. [3] was developed in order to have a number of advantages over the traditional approach based on vectors and Euler angles. Firstly, in the traditional approach, as the moment vectors are computed in the LCS, the method involves multiple successive coordinate transformations. Dumas et al. characterized the method as a 3 step process (and from this point this methodology will be referred to as the 3 step method). In contrast, employing wrench notation allows all calculations to be performed in the GCS, facilitating a 1 step process (the 1 step method). This is advantageous as it eliminates the need for multiple coordinate transformations. Secondly, the 3 step method is normally computed using Euler angles, and thus is subject to the problem of gimbal lock, that is the existence of singularities in certain body postures. In contrast, employing unit quaternions to parameterize rotation eliminates these singularities.

Dumas et al. [2] studied the impact of the choice of inverse dynamics method on the calculation of kinematics and inter-segmental joint forces and moments during gait. They found that the choice of method had little impact on the calculation of kinematics. However, the impact of the choice of method on the computation of kinetics was profound. They demonstrated that the choice of inverse dynamics methodology had little impact upon forces or moments at the ankle, but that the effects were large at both the knee and the hip and of particular significance during the swing phase of gait. These results are startling. Although the computational frameworks vary, the methods themselves are derived from classical mechanics and are therefore theoretically equivalent. In particular, Dumas et al. suggested that the dynamics computation predicated the observed differences. Further work needs to be performed to ascertain the nature of the differences between the methods and to provide some guidelines for the appropriate choice of methodology. In performing sensitivities concerning the effect of the dynamics computation it is important to harmonize the assumptions as closely as possible. A key difference between the implementation of the 1 and 3 step methods in the work of Dumas et al. is the choice of rotation parameterization. Although the authors chose a rotation computation based on Euler angles and rotation matrices based on the typical inverse dynamics implementations this is not a necessary stipulation of the method. The purpose of this study was therefore to compare the inter-segmental moments calculated by the 1 and 3 step methods when the kinematics procedures of both methods were based on a quaternion parameterization of rotation and thus the effect of the dynamics computation alone could be explored.

## Methods

**Figure 1:**
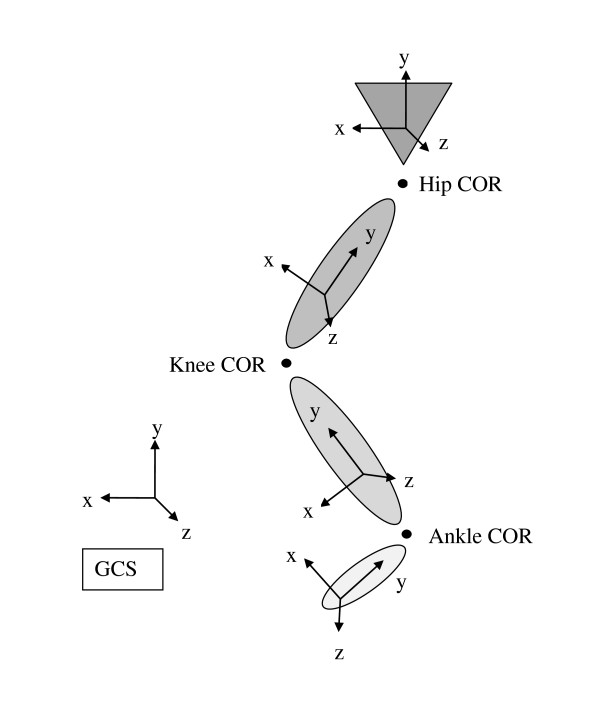
**A sketch of the musculoskeletal model employed in this study**.

In this study, a previously described musculoskeletal model of a right lower limb [5] was employed in order to evaluate the effect of the choice of inverse dynamics methodology on the calculation of inter-segmental moments during jumping and weightlifting movements. The musculoskeletal model was a linked rigid segment model consisting of four segments (foot, calf, thigh and pelvis), articulated by three ball and socket joints at the ankle, knee and hip (Figure 1). The model therefore permitted 3D rotation but precluded joint translations.

### Experimentation

The data set was acquired using the VICON motion capture system (Vicon MX System, Vicon Motion Systems Ltd, Oxford, UK) to establish the position of reflective markers placed on key anatomical landmarks [6,7] and a Kistler portable force plate (Kistler Type 9286AA, Kistler Instrumente AG, Winterthur, Switzerland) to measure ground reaction force. In each movement trial, only the subject's right foot was placed on the force plate, thus the analysis was for the right limb alone. All movement data was collected at 200 Hz. The ground reaction force recorded from the force plate was considered to act at the distal end of the foot segment, and thus the centre of pressure measurement was used to calculate the moment acting at the distal end of the foot.

The subjects were 11 athletic males who provided informed consent (mean age 26.6 years; mean mass 83.6 kg). After performing a standardised warm up, each subject performed a series of 5 maximal vertical jumps - the highest of which was chosen for analysis. Of the 11 subjects, 10 subjects (mean age 26.7 years; mean mass 84.7 kg) were familiar with the push jerk exercise (where the barbell is dynamically jerked over head using the legs) and thus performed this exercise with 40 kg following the vertical jump. Finally, 4 subjects (mean age 28.5 years; mean mass 80.1 kg) also performed a body weight squat to a self selected depth.

The raw data set consisted of the position of the markers and the ground reaction force for the right foot during each activity. The raw position of the markers was transformed into the translations and rotations that represented the position and orientation of the each segment of the model using the method of Horn [8]. Finally, these parameters were smoothed using the linear time-invariant and quaternion filtering techniques described by Lee and Shin [9]. The data sets were then analyzed using two distinct inverse dynamics techniques - the 1 step method employing unit quaternions and wrench notation described by Dumas et al. [3] and a traditional 3 step approach [1,10] employing a quaternion parameterization of rotation. In order to best contrast the 2 different methods, both the calculation of the kinematic quantities and the dynamics computation were varied between the methods. In order to make the two methods as similar as possible the COM of each segment was specified to be on the longitudinal axis of the segment and at a given ratio of the proximal to distal length taken from the anthropometric model [11]. Thus although the position of the COM of each segment was defined by a vector in the 1 step method, in this implementation this vector specified the same position as for the 3 step method.

### Kinematics - 1 Step Method

Given the vectors ***r****_i _*and ***p****_i _*representing the position in the GCS of the COM and the proximal joint of a segment respectively, the vector $C_{i}^{s}$ representing the vector from the proximal joint to the COM in the LCS and the unit quaternion ***q ***representing the transformation from the LCS to the GCS, then [3]:

(1) $$\begin{array}{l} \left[\begin{array}{l} 0\\ r_{i} \end{array}\right]=\left[\begin{array}{l} 0\\ p_{i} \end{array}\right]+q_{i}⊗\left[\begin{array}{l} 0\\ C_{i}^{s} \end{array}\right]⊗q_{i}^{*}\\ \Rightarrow \left[\begin{array}{l} 0\\ {\dot{r}}_{i} \end{array}\right]=\left[\begin{array}{l} 0\\ {\dot{p}}_{i} \end{array}\right]+{\dot{q}}_{i}⊗\left[\begin{array}{l} 0\\ C_{i}^{s} \end{array}\right]⊗q_{i}^{*}+q_{i}⊗\left[\begin{array}{l} 0\\ C_{i}^{s} \end{array}\right]⊗{\dot{q}}_{i}^{*}\\ \Rightarrow \left[\begin{array}{l} 0\\ \alpha_{i} \end{array}\right]=\left[\begin{array}{l} 0\\ {\ddot{r}}_{i} \end{array}\right]=\left[\begin{array}{l} 0\\ {\ddot{p}}_{i} \end{array}\right]+{\ddot{q}}_{i}⊗\left[\begin{array}{l} 0\\ C_{i}^{s} \end{array}\right]⊗{\ddot{q}}_{i}^{*}\\ +2\left({\dot{q}}_{i}⊗\left[\begin{array}{l} 0\\ C_{i}^{s} \end{array}\right]⊗{\dot{q}}_{i}^{*}\right)+q_{i}⊗\left[\begin{array}{l} 0\\ C_{i}^{s} \end{array}\right]⊗{\ddot{q}}_{i}^{*} \end{array}$$

where the linear acceleration of the COM $a_{i}={\ddot{r}}_{i}$. The segment angular velocity $\omega_{i}=\left[\omega_{x_{i}},\omega_{y_{i}},\omega_{z_{i}}\right]$in the GCS was given by [3]:

(2) $$\left[\begin{array}{l} 0\\ \omega_{i} \end{array}\right]=2{\dot{q}}_{i}q_{i}^{*}$$

and the segment angular acceleration $\alpha_{i}=\left[\alpha_{x_{i}},\alpha_{y_{i}},\alpha_{z_{i}}\right]$in the GCS by [3]:

(3) $$\left[\begin{array}{l} 0\\ \alpha_{i} \end{array}\right]=2\left({\ddot{q}}_{i}q_{i}^{*}+{\dot{q}}_{i}{\dot{q}}_{i}^{*}\right)$$

Finally, the inertia tensor ***I****_i _*in the LCS was taken from the anthropometric model [10] and transformed into the GCS, where [3]:

(4) $$I_{i}=R_{i}I_{i}^{s}R_{i}^{-1}$$

and where the axes of the LCS were taken to correspond to the principal axes of the segment such that:

$$I_{i}=\left[\begin{array}{ccc} Ix_{i} & 0 & 0\\ 0 & Iy_{i} & 0\\ 0 & 0 & Iz_{i} \end{array}\right]$$

and ***R ***is the rotation matrix from the LCS to the GCS given by [12]:

(5) $$R=\left[\begin{array}{ccc} q_{0}^{2}+q_{1}^{2}-q_{2}^{2}-q_{3}^{2} & 2\left(q_{1}q_{2}-q_{0}q_{3}\right) & 2\left(q_{1}q_{3}+q_{0}q_{2}\right)\\ 2\left(q_{2}q_{1}+q_{0}q_{3}\right) & q_{0}^{2}-q_{1}^{2}+q_{2}^{2}-q_{3}^{2} & 2\left(q_{2}q_{3}-q_{0}q_{1}\right)\\ 2\left(q_{3}q_{1}-q_{0}q_{2}\right) & 2\left(q_{3}q_{2}+q_{0}q_{1}\right) & q_{0}^{2}-q_{1}^{2}-q_{2}^{2}+q_{3}^{2} \end{array}\right]$$

### Kinematics - 3 Step Method

Firstly, the position of the COM in the GCS was found by evaluating the position of the COM in the LCS coordinate frame, then transforming this position into the GCS. The linear acceleration of the COM was then found by direct numerical differentiation of the COM position. This was found to yield an equivalent result to the 1 step approach (which would be expected as the methods are analytically the same). Secondly the angular velocity ***ω****^S ^*in the LCS was found using the identity:

(6) $$\left[\begin{array}{l} 0\\ \omega_{i}^{s} \end{array}\right]=2q_{i}^{*}{\dot{q}}_{i}$$

Thirdly, in this case the angular acceleration in the LCS was found by direct numerical differentiation of the angular velocity. Again, this was found to yield a similar result to the analytical approach employed in the 1 step method.

### Inverse Dynamics - 1 Step Method

**Figure 2:**
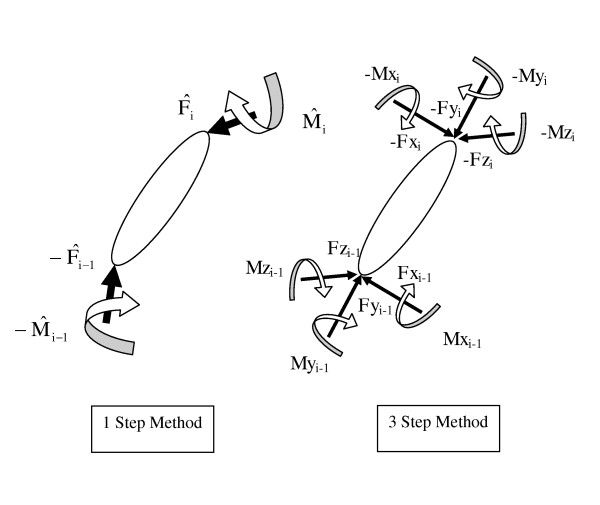
**Definition of forces and moments for the 1 and 3 step methods**. Note the different sign convention employed in the two methods.

The 1 step method was based upon representing the forces and moments at a joint by a wrench; a 6 D vector where the first three terms represent the force vector and the second three terms the moment vector. The Newton-Euler equations could then be expressed by the following equation [3]:

(7) $$\begin{array}{l} \left[\begin{array}{l} F_{i}\\ M_{i} \end{array}\right]=\left[\begin{array}{cc} m_{i}E_{3\times 3} & 0_{3\times 3}\\ m_{i}{\tilde{c}}_{i} & I_{i} \end{array}\right]\left[\begin{array}{c} \alpha_{i}-g\\ \alpha_{i} \end{array}\right]\\ +\left[\begin{array}{c} 0_{3\times 1}\\ \omega_{i}\times I_{i}\omega_{i} \end{array}\right]+\left[\begin{array}{cc} E_{3\times 3} & 0_{3\times 3}\\ {\tilde{d}}_{i} & E_{3\times 3} \end{array}\right]\left[\begin{array}{c} F_{i-1}\\ M_{i-1} \end{array}\right] \end{array}$$

where:

***F****_i _*= [*Fx_i_, Fy_i_, Fz_i_*] - proximal joint reaction forces (see Figure 2)

***F***_*i-*1 _= [*Fx*_*i*-1_, *Fy_i_*-1, *Fz*_*i*-1_] - distal joint reaction forces (see Figure 2)

***M****_i _*= [*Mx*_*i*_, *My_i_, Mz_i_*] - proximal joint moments (see Figure 2)

***M****_i_*_-1 _= [*Mx _i_*_-1_, *My _i_*_-1_*, Mz _i_*_-1_] - distal joint moments (see Figure 2)

*m_i _*- segment mass

***E***_3×3 _- identity matrix

**0**_3×3_, **0**_3 × 1 _- zero matrix, vector

***C****_i _*- vector from the proximal joint to the segment COM

***d****_i_*- vector from the proximal to the distal joint

***g ***- acceleration due to gravity

and $\tilde{c},\tilde{d}$ represented the skew symmetric matrix of a 3D vector such that:

(8) $$\tilde{c}=\left[\begin{array}{ccc} 0 & -c_{3} & c_{2}\\ c_{3} & 0 & -c_{1}\\ -c_{2} & c_{1} & 0 \end{array}\right],\tilde{d}=\left[\begin{array}{ccc} 0 & -d_{3} & d_{2}\\ d_{3} & 0 & -d_{1}\\ -d_{2} & d_{1} & 0 \end{array}\right]$$

Equation 7 was therefore applied to each joint in sequence, moving from distal to proximal, in order to evaluate the forces and moments in the GCS.

### Inverse Dynamics - 3 Step Method

The first step in the iteration was to calculate the inter-segmental forces for each frame. Forces were calculated by consideration of the free body diagram (see Figure 2) of each segment, iteratively, moving proximally along the kinetic chain. Next, the moment at each joint was found by employing Euler's equations of 3D motion for a rigid body sequentially, again by moving proximally along the lower limb.

In order to employ the Euler equations all variables were first transformed into the LCS of the relevant segment. In this case the rotational equations of motion about the COM of the segment could be employed as the rotation from GCS to LCS is independent of reference point [1,9,11]:

(9) $$\begin{array}{l} Ix_{i}\alpha_{xi}+\left(Iz_{i}-Iy_{i}\right)\omega_{yi}\omega_{zi}=\\ -Fz_{i-1}l_{di}-Fz_{i}l_{pi}+Mx_{i-1}-Mx_{i}\\ Iy_{i}\alpha_{yi}+\left(Ix_{i}-Iz_{i}\right)\omega_{xi}\omega_{zi}=My_{i-1}-My_{i}\\ Iz_{i}\alpha_{zi}+\left(Iy_{i}-Ix_{i}\right)\omega_{xi}\omega_{yi}=\\ Fx_{i-1}l_{di}+Fx_{i}l_{pi}+Mz_{i-1}-Mz_{i} \end{array}$$

where *l_pi _*and *l_di _*represent the distance from COM to the proximal and distal joints, respectively. For each segment, the calculated moment was then transformed back into the GCS, in order for it to then be transformed into the LCS of the proximal segment to be used as an input in the next stage of the iteration.

## Results

**Figure 3:**
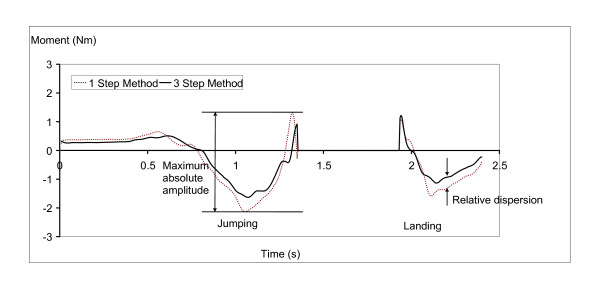
**A graph illustrating the definition of maximum absolute amplitude and relative dispersion**.

There were two methods of comparing inter-segmental moments. Firstly, the differences between the peak moments predicted by the two methodologies were compared using paired t-tests where alpha was set to 0.05 a priori. Secondly, the ratio *r *was calculated using the methodology of Dumas et al. [2] where the ratio *r *simply represents the maximum dispersion of the curves normalized by the maximum amplitude of the curves. The maximum relative dispersion was the largest difference between the two curves at a given time. The maximum amplitude of the curves was defined to be the difference between the largest and the smallest value based on either method. These values are depicted on Figure 3.

The two different methods yielded very similar outputs in terms of the kinematics and inter-segmental forces calculated during the movements studied. In contrast to the work of Dumas et al. [2] the inter-segmental moments observed during the two activities were also found to be in close agreement. The mean values of the peak moments were identical to at least 2 decimal places in all cases and were generally not statistically different (p > 0.05 for 54 of 72 comparisons). On an individual basis, the mean absolute percentage difference between the peak moments calculated (when considering all three planes) was 0.1% for jumping, 0.2% for landing, 0.1% for jerking and 0.1% for squatting.

Table 1 presents the mean ratio *r *of the two methods in the frontal plane (moment about the x-axis), the transverse plane (moment about the y-axis) and the sagittal plane (moment about the z-axis) for each of the studied activities. The small *r *values show that there are very few differences between the two methods. The differences that exist are magnified at the more proximal joints as *r *was greater at the knee, and largest at the hip. The choice of inverse dynamics method had the smallest effect during squatting and was most influential during jumping.

**Table 1:** Mean ratio *r *(in%) between the maximal relative dispersion and the maximal absolute amplitude.

Activity	Ankle			Knee			Hip		
	x-axis	y-axis	z-axis	x-axis	y-axis	z-axis	x-axis	y-axis	z-axis
Jump	0.00 ± 0.00	0.02 ± 0.01	0.00 ± 0.00	0.46 ± 0.22	0.29 ± 0.18	0.07 ± 0.03	1.72 ± 0.64	0.63 ± 0.25	0.21 ± 0.09
Land	0.00 ± 0.00	0.03 ± 0.02	0.00 ± 0.00	0.13 ± 0.06	0.21 ± 0.09	0.05 ± 0.03	0.67 ± 0.35	0.52 ± 0.25	0.13 ± 0.06
Jerk	0.00 ± 0.00	0.01 ± 0.00	0.00 ± 0.00	0.06 ± 0.03	0.06 ± 0.04	0.01 ± 0.00	0.20 ± 0.08	0.15 ± 0.06	0.06 ± 0.02
Squat	0.00 ± 0.00	0.00 ± 0.00	0.00 ± 0.00	0.01 ± 0.00	0.00 ± 0.00	0.00 ± 0.00	0.03 ± 0.02	0.04 ± 0.02	0.01 ± 0.00

Data sets include jumping (n = 11), landing (n = 11), jerking (n = 10) and squatting (n = 4), and are presented in each of the 3 planes of movement: frontal plane (moment about x-axis), transverse plane (moment about y-axis) and sagittal plane (moment about z-axis).

## Discussion

In this study, two different inverse dynamics methodologies were used to calculate kinematics and inter-segmental forces and moments during vertical jumping, jerking and squatting. Two different methodologies for calculating the kinematics were employed, both of which were based upon a quaternion description of rotation. The two different methodologies yielded markedly similar results. The first methodology (employed in the 1 step method) involved deriving an analytical solution for the linear acceleration of the COM, and the angular velocity and acceleration of the segment in terms of the unit quaternion describing the rotation and its derivatives. The kinematics were then computed directly from these expressions. In contrast, the second methodology (employed in the 3 step method) involved first calculating the position of the COM and the angular velocity of the segment using quaternion relationships. Higher derivatives of these quantities (in particular, the linear acceleration of the COM and the angular acceleration) were then calculated by direct numerical differentiation of these variables. That these two distinct methods yielded the same result is unsurprising as the approaches are analytically the same, however, the results of this study do confirm that the two approaches are also computationally equivalent. Similarly, the finding that inter-segmental forces did not vary between the two methods is to be expected as the two methodologies employ the same approach to calculating joint forces.

In contrast to the previous work of Dumas et al. [2] in this study the choice of inverse dynamics method had very little effect on the calculation of inter-segmental moments. This result is intuitively appealing. Although the computation of joint moments is different, both methodologies are derived from a consideration of classical mechanics and are thus theoretically equivalent. Although small differences between the methods might be expected due to computational differences, the large divergences found by Dumas et al. seem to suggest fundamental differences between the methods.

Dumas et al. [2] report large deviations between the methods in terms of the inter-segmental forces. In the inverse dynamics method, inter-segmental forces are calculated simply by considering the net force on each segment in combination with the linear acceleration of the COM of the segment. Given that the force on the distal end of the lower limb was constant between the 1 and 3 step methods it seems likely that differences in the inter-segmental forces are due to differences in the accelerations of the segment COMs. The veracity of this assumption is hard to ascertain from the work of Dumas et al. as the only kinematic data included in their work is the angular velocity of the segments, although they observed that differences in kinematics were limited.

It is clear that the differences in inter-segmental forces provide the most likely explanation for the differences in inter-segmental moments found in the work of Dumas et al. [2]. Certainly the results of the present work support this contention with regards to 1 and 3 step methods, as given similar inter-segmental forces, both yielded similar inter-segmental moments, implying that there are no differences in the dynamics computation of the inter-segmental moments. Dumas et al. concluded that the observed differences in their work were due to the dynamic computation. In contrast, the findings of this study indicate that there are no meaningful differences between the methods in terms of the dynamics computation. Instead the authors of this study tentatively believe that the differences seen in the work of Dumas et al. are more likely to be caused by differences in the kinematics, contrary to Dumas et al.'s own suggestions.

It is worth noting that the work of Dumas et al. [2] is based upon a study of gait, whereas the present study considers vertical jumping. However the authors of the present work do not believe that the choice of activity is important with regards to the present conclusions. In this paper it is contended that the dynamics computations do not differ markedly. If a different activity were chosen, the forces and moments input to the dynamics computation will not differ between the methods. Instead, the only possible source of variation would be in the kinematics. Further work is therefore required to understand the sensitivity of kinematics computation to rotation parameterizations.

## Conclusions

In summary, this study is contrary to the findings of Dumas et al. [2] in finding little influence of the choice of inverse dynamics methodology on the calculation of inter-segmental joint moments, thus the methods can be used interchangeably. There are a number of advantages to the 1 step approach. These include computational efficiency and the elegance of the quaternion formalism. For these reasons the authors of this paper support the contention of Dumas et al. that a 1 step approach may, in many applications, represent the method of choice.

The findings of this study are particularly important in the light of previous evidence that had suggested that the dynamics computation for the 1 and 3 step method differed. Future work should seek to compare the alternate methods of inverse dynamics identified by Dumas et al. which have also been shown to produce diverging solutions. These studies should aim to harmonize assumptions that are not fundamental to the basis of the methods in order to establish if the observed differences are truly the result of the dynamics computation. Equally, further work is needed to establish the sensitivity of kinematics computations to the choice of rotation parameterization.

## Competing interests

The authors declare that they have no competing interests.

## Authors' contributions

DJC collected the data, created the musculoskeletal model employed, analyzed the data and drafted the manuscript. AMJB participated in the design and construction of the musculoskeletal model, and in the design of the data collection and analysis procedures. Both authors read and approved the final manuscript.
